# A tissue-engineered urinary conduit in a porcine urinary diversion model

**DOI:** 10.1038/s41598-021-94613-7

**Published:** 2021-08-18

**Authors:** Arkadiusz Jundziłł, Piotr Kwieciński, Daria Balcerczyk, Tomasz Kloskowski, Dariusz Grzanka, Paulina Antosik, Katarzyna Meger, Marta Pokrywczyńska, Tomasz Drewa

**Affiliations:** 1 Department of Regenerative Medicine, Cell and Tissue Bank, Collegium Medicum in Bydgoszcz, Nicolaus Copernicus University in Torun, Skłodowskiej-Curie 9, 85-094 Bydgoszcz, Poland; 2 Department of Plastic, Reconstructive and Aesthetic Surgery, Collegium Medicum in Bydgoszcz, Nicolaus Copernicus University in Torun, Bydgoszcz, Poland; 3 Veterinary Clinic Vet-Lab Brudzew, Brudzew, Poland; 4 Department of Clinical Pathomorphology, Collegium Medicum in Bydgoszcz, Nicolaus Copernicus University in Torun, Bydgoszcz, Poland; 5 Medical Equipment Producer Galmed, Bydgoszcz, Poland

**Keywords:** Bladder cancer, Regenerative medicine, Tissue engineering

## Abstract

The use of an ileal segment is a standard method for urinary diversion after radical cystectomy. Unfortunately, utilization of this method can lead to numerous surgical and metabolic complications. This study aimed to assess the tissue-engineered artificial conduit for urinary diversion in a porcine model. Tissue-engineered tubular polypropylene mesh scaffolds were used for the right ureter incontinent urostomy model. Eighteen male pigs were divided into three equal groups: Group 1 (control ureterocutaneostomy), Group 2 (the right ureter-artificial conduit-skin anastomoses), and Group 3 (4 weeks before urostomy reconstruction, the artificial conduit was implanted between abdomen muscles). Follow-up was 6 months. Computed tomography, ultrasound examination, and pyelogram were used to confirm the patency of created diversions. Morphological and histological analyses were used to evaluate the tissue-engineered urinary diversion. All animals survived the experimental procedures and follow-up. The longest average patency was observed in the 3rd Group (15.8 weeks) compared to the 2nd Group (10 weeks) and the 1st Group (5.8 weeks). The implant’s remnants created a retroperitoneal post-inflammation tunnel confirmed by computed tomography and histological evaluation, which constitutes urostomy. The simultaneous urinary diversion using a tissue-engineered scaffold connected directly with the skin is inappropriate for clinical application.

## Introduction

Despite the intensive development of regenerative medicine and technological progress, incontinent urinary diversion (UD), including ureterocutaneostomy or ureteroileocutaneostomy, are dominant treatment methods^1^. Nowadays, the ileal conduit constitutes, despite apparent disadvantages, the preferable method after cystectomy treatment^2^. The ileal segment is also a standard UD method after radical cystectomy in elderly patients^3^. A decreasing number of bowel wall neobladder diversions is correlated with two factors: aging patients and expectation for livelong good quality of life^4^. Another reason is the fear of perioperative complications resulting from the surgery’s length and the digestive tract’s violation^5–7^. Moreover, a reduced number of orthotopic diversion procedures participate with intensive minimally invasive surgical techniques development^8^. The intensive development of cost-effectiveness robotic surgery forces a new UD method without employing the digestive tract.

Many preclinical studies are assessing the usefulness of tubular structures in urinary tract reconstructive urology. However, studies showed that conduit is a junction between the ureter and the skin with the most value, mimicking the encountered clinical situations^9^.

Such estimation of artificial conduit (AC) was initiated by Drewa et al. using small intestinal submucosa (SIS) seeded with 3T3 fibroblasts for UD in a rat model^10^. The congruous rodent model of UD was used for appraisal bladder acellular matrix (BAM) seeded with urothelial cells (UC)^11^. Then in 2014, Kloskowski et al. reported partial successful UD using acellular aortic arch (AAM) and electrospun nanofibrous scaffold made of synthetic poly(l-lactide-co-caprolactone) (PLCL)^12^. Another evaluation of tissue-engineered tubular construct: BAM seeded with Adipose-Derived Stem Cells (ADSCs), and Smooth Muscle Cells (SMCs) cell with promising results was reported by Meng et al.^13^.

The evaluation of the porcine UD model in assessing the utilization of an AC in the preclinical application in large animal models was conducted by Gentjes et al.^14^ and Basu et al.^15^, both in 2012. These two preclinical studies used Collagen (type I), and Vypro mesh seeded with UC and polyglycolic acid (PGA) coated with PLGA seeded with SMCs, respectively^15^. Notwithstanding, none of them used the AC preimplantation for better integration with host tissues before ureter reconstruction. Sloff et al. performed preimplantation of the cellular construct, but results confirmed integration with bladder dome, not recalling skin-conduit-ureter connection^16^.

We have no clear answer to what type of biomaterial is the best for tubular construct creation for UD^16,17^. Moreover, contrary to previous studies, our AC is not urine resistant. That is why, in this study, we first utilize the preimplantation before the ureter reconstruction model in the UD model. We compared the results with the AC urinary tract diversion model and ureterocutaneostomy, which has not been performed yet.

## Methods

### Polymer scaffolds

**Figure 1:**
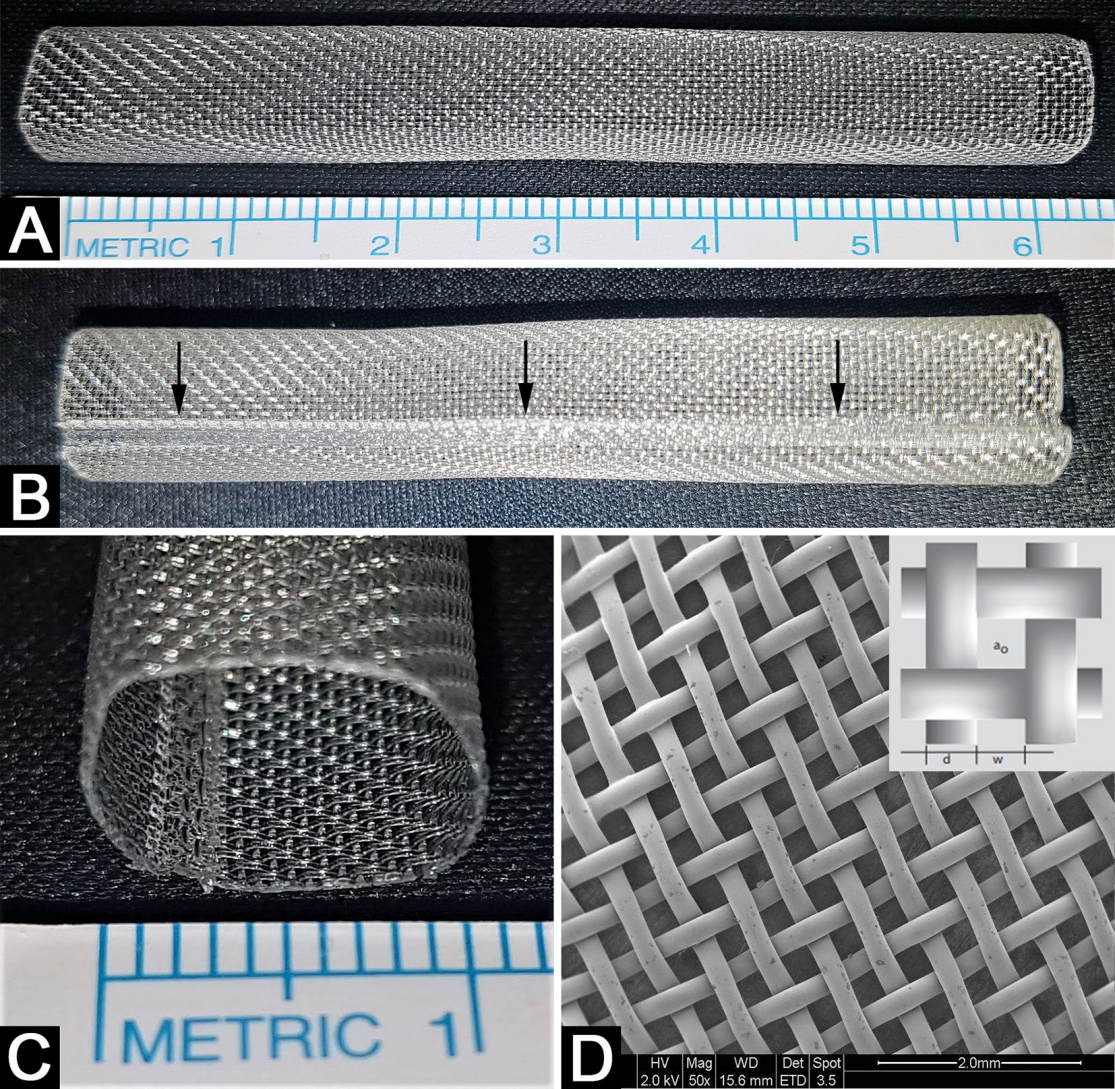
Artificial conduit characterization. (**A**) The mesh is woven synthetic monofilament open propyltex mesh fabrics. (**B**) The ultrasonic welding machine was used to produce tubular 3D construct; arrows indicate the weld formation. (**C**) Close look on tube cross-section. (**D**) Scanning Electron Microscopy, bar 2 mm., mag ×50. The characteristics of one grid module: a_o_—34 n/cm, d—215 µm, w—297 µm.

Tubular constructs 60 mm long and 10 mm in diameter were created from polypropylene mesh sheets using an ultrasonic welding machine for tube creation. Then they were sterilized in ethylene oxide. Scanning electron microscopy was utilized for tubular conduit material and weld evaluation (Fig. 1).

### Mesh characterization

The mesh is woven synthetic monofilament open propyltex mesh fabrics. Mesh warp and weft count (n) were both 20 n/cm. The weight 125 g/m^2^ was assessed by the DIN53854 method. The thickness of the mesh was 420 µm measured by DIN 538555. Specific gravity was 0.91 g/mm^3^. The tensile strength was evaluated in a range of 35–62 daN/mm^2^. The mesh elongation at the break was between 20 and 50%. The material passes USP plastics class VI tests. Raw materials comply with FDA 21 CFR 178. The propyltex is biocompatible according to ISO 10993-ff. Precision monofilament fabrics have defined surface characteristics with the validated production process following EU-GMP guidelines. The mesh was fabricated in a cleanroom class 7 (ISO 14644-1).

### Construction of tissue-engineered urinary conduit (pre-implanted)

The tubular mesh construct was implanted intramuscularly for 4 weeks. The inset was placed intramuscularly at an angle of 45° to the abdomen wall. The region of conduit preimplantation was precisely matched for not change its place during subsequent reconstruction. We inserted part of the endotracheal tube 5.5 F for lumen preservation, which protruded by 1 cm on each side of the tube.

### Implantation in the urostomy model

**Figure 2:**
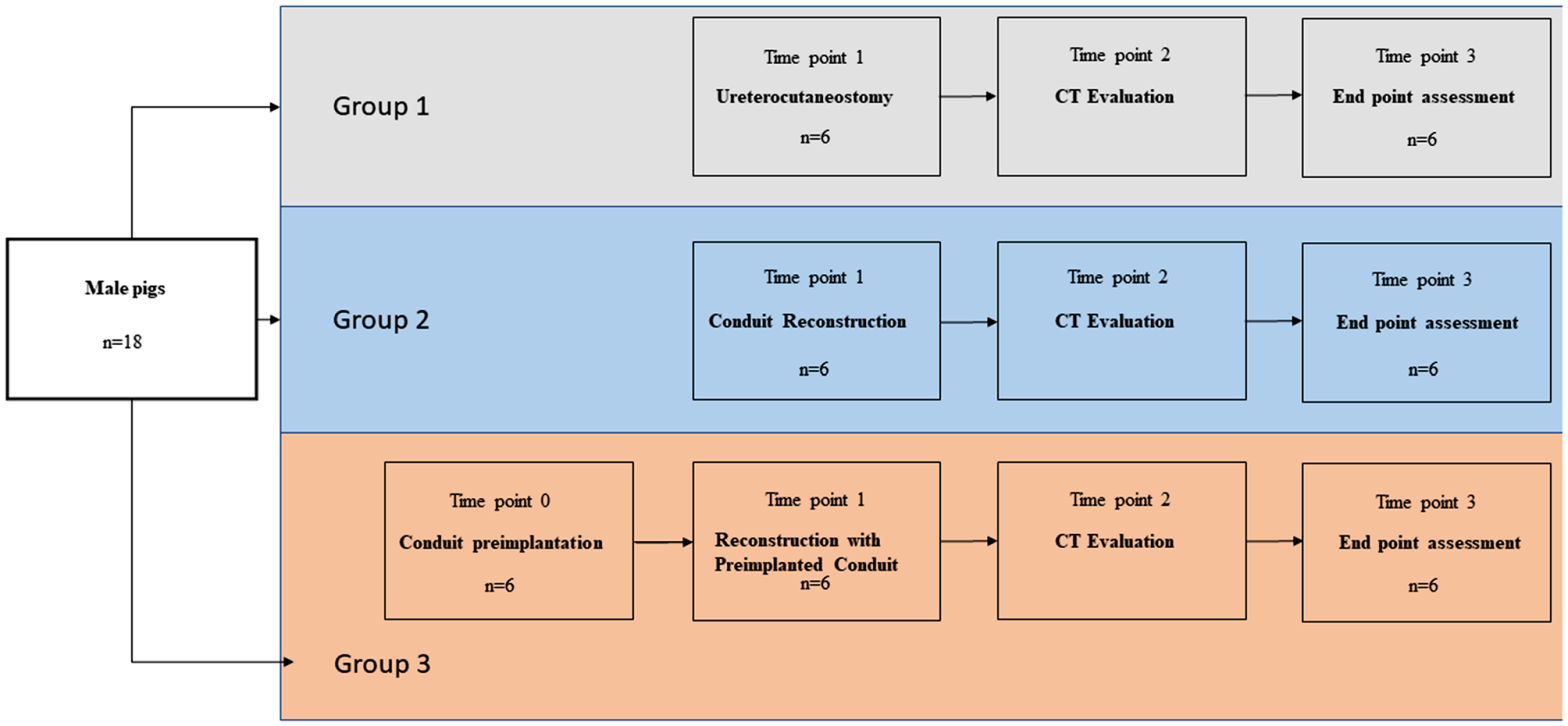
Diagram of the conducted experiment. Timepoint 0—conduit preimplantation 4 weeks before urostomy reconstruction—Group 3, Timepoint 1—urostomy creation, Timepoint 2—(7–8 weeks post urostomy formation), Timepoint 3–6 months post urostomy reconstruction.

We used 18 male Landrace pigs weighing about 60 kg each. The animals were divided into three equal groups, with 6 per each. Tissue-engineered tubular scaffolds were used for the construction of the artificial urinary conduits despite the control group. Conduits were implanted as an incontinent urostomy using right ureters. In the first Group—the control group—an ureterocutaneostomy was created (n = 6). In the 2nd Group, the right ureter-artificial conduit-skin anastomoses (n = 6) (the AC model) were performed without preimplantation. In the 3rd Group (n = 6) 4 weeks before urostomy, the artificial conduits were preimplanted before reconstruction between abdomen muscles (Fig. 2). This experiment was approved, and all procedures were performed under the Committee’s agreement on the Ethics of Animal Experiments of the University of Technology, and Life Science in Bydgoszcz, Poland (no. 43/2018). This study was carried out in compliance with the ARRIVE guidelines. Animals were housed individually with a restricted diet. Free access to an automatic tap water system was assured.

### Surgical procedure

**Figure 3:**
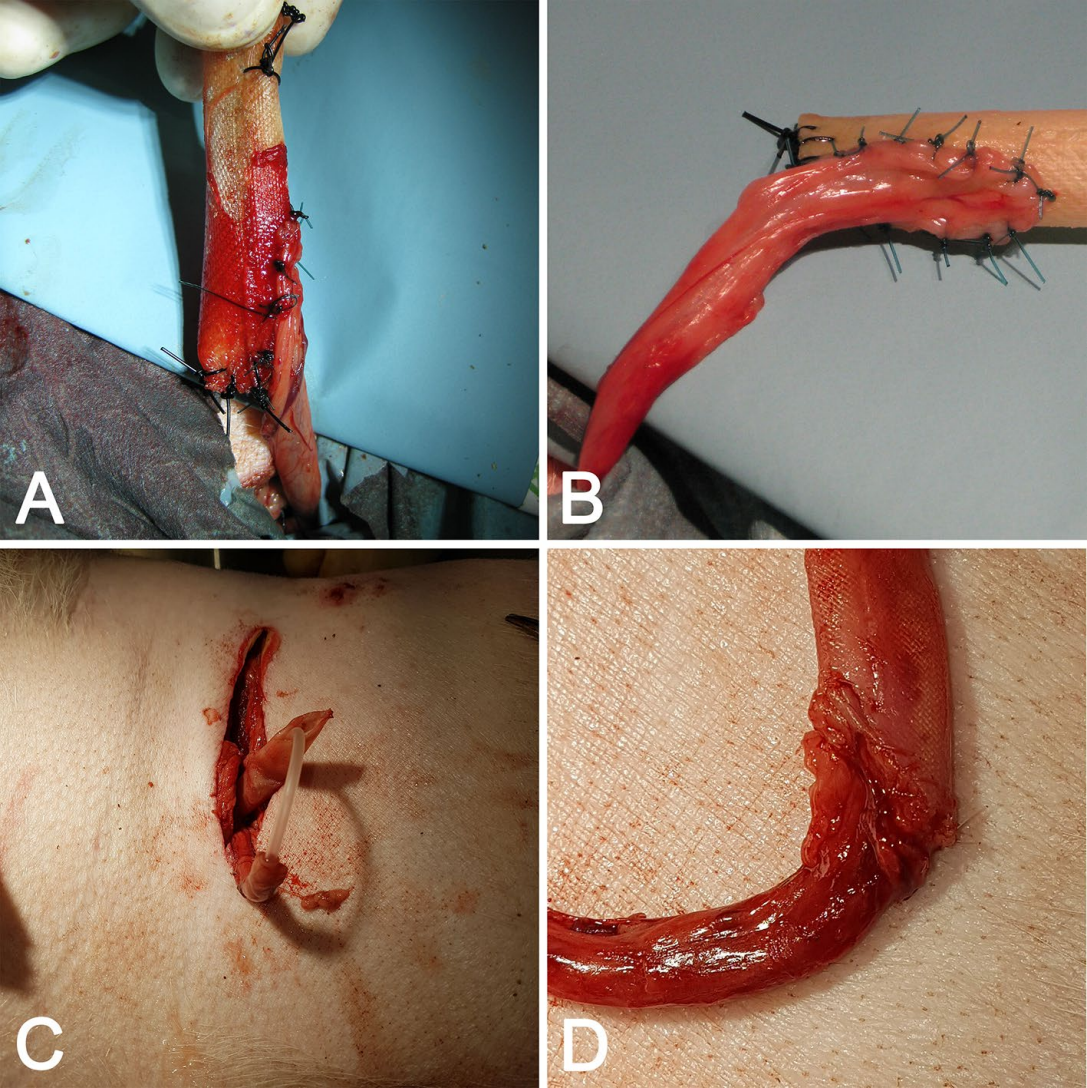
Method of side-by-side uretero-conduit anastomosis. (**A**,**B**) The anastomosis between AC and ureter from different point of view-Group 2, (**C**,**D**) Group 3—pre-implanted and prepared AC for anastomosis by trimmed tip, (**D**) AC anastomosed with ureter.

**Figure 4:**
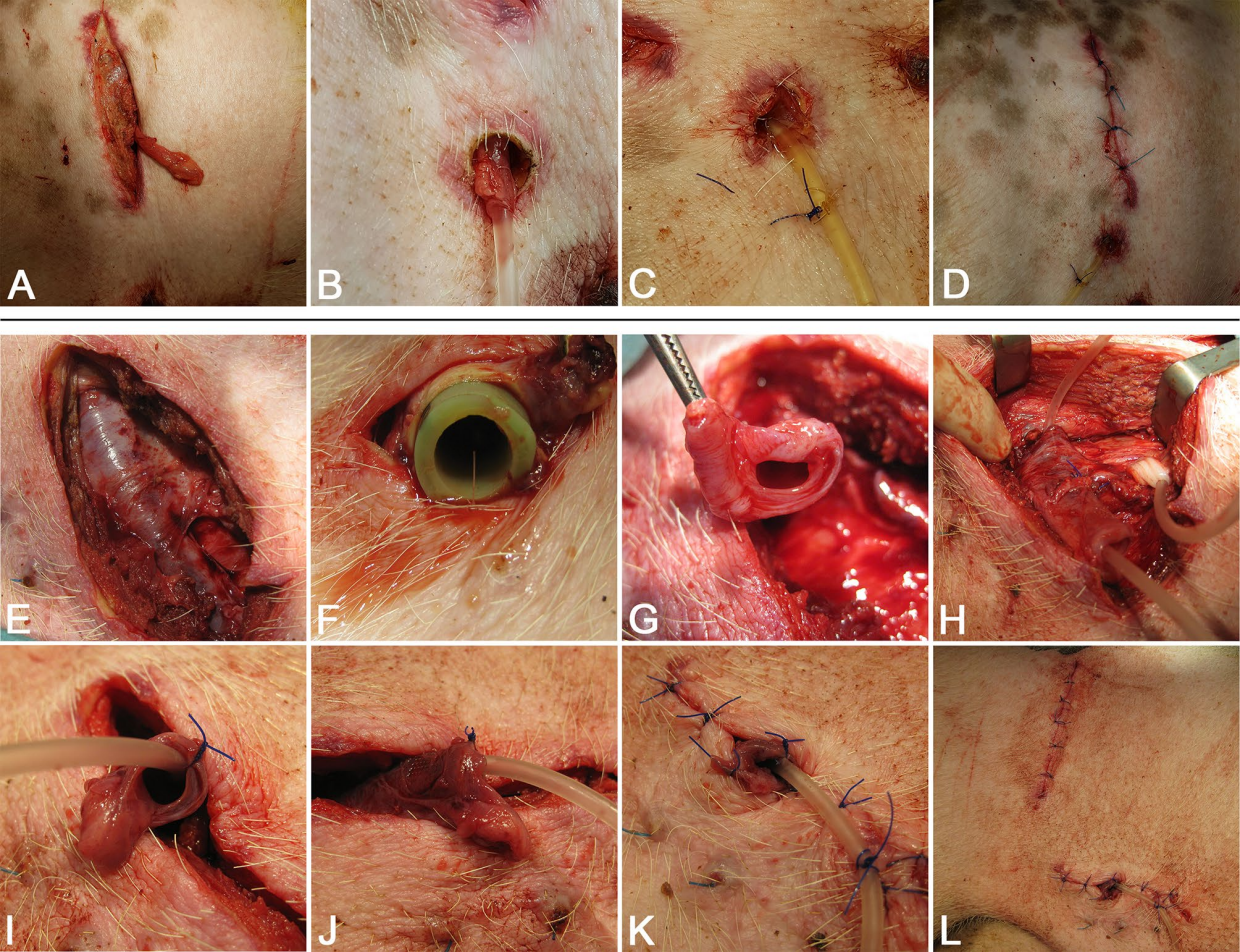
Surgical procedures. Ureterocutaneostomy formation in Control Group 1 (**A**–**D**) (**A**,**B**) the proximal part of a right ureter is exposed, (**C**) the ureter was stented with 10 F Couvelaire catheter, (**D**) ureterostomy was planned 3 cm below surgical access before right hind limb, around 1 cm in diameter skin defect were performed. The ureter was connected to the skin by interrupted 4.0 Monocryl sutures; the catheter was attached to the skin with 3.0 Nylon sutures. Urostomy reconstruction in preimplantation conduit before reconstruction-Group 3 (**E**–**H**): (**E**) a month after scaffold preimplantation, (**F**) remove the tube from preimplanted conduit, (**G**) conduit ready for reconstruction, (**H**) Couvelaire catheter pulled through the conduit and inserted to the spatulated ureter, (**I**) after anastomosis side-to-side with preimplanted conduit, (**J**) preimplanted conduit has been sewn to the skin with nonabsorbable 4.0 with 6–8 individual stitches, (**K**) Couvelaire catheter was attached to the skin with a nonabsorbable suture, (**L**) state post urostomy reconstruction with preimplanted conduit.

After general anesthesia, a lateral incision was made. Delaminating the abdomen wall muscles, we entered the retroperitoneal cavity, leave the intraperitoneal cavity intact. Then the right ureter was exposed, mobilized in the bladder direction, and transected near the bladder wall. The ureter distal stump was ligated preventively. The proximal part of the ureter following the kidney was undisturbed, caused by the fear of lost blood supply. Ureterostomy was performed 3 cm below surgical access before the hind leg. The urine passage led through round 1 cm diameter skin excision, cut fascia, and stratified muscles. Before reconstruction, the ureter was stented with 10 or 12 F Couvelaire ureteral catheters fixed to the skin with nonabsorbable 3.0 Nylon sutures (Fig. 3). In the 1st Group (control), the ureter was connected to the skin by interrupted 4.0 Monocryl sutures. In the 2nd Group and 3rd Group (4 weeks after preimplantation), the ureter was spatulated and sutured side-to-side with AC using Monosyn 4.0 running suture. For side-to-side anastomosis, the AC had to be appropriately prepared. One side was cut at an angle of 45° that created a hole that coincides with the lumen of the spatulated ureter (Fig. 3). The conduit was attached to the fascia with 4.0 PDS sutures. The conduit was attached at an angle of 45 to the skin. The conduit has been sewn to the skin with nonabsorbable 4.0 with 6–8 individual stitches. The wound was closed in layers: delaminated muscles were brought together with 4.0 Monosyn interrupted sutures, fascia with simple continuous pattern with PDS 2.0 loop, skin with single 2.0 Dafilon seams (Fig. 4). Catheters introduced into reconstructed ureters were maintained for 3 weeks on a follow-up period. Perioperative antibiotic therapy was administered for 7 days.

### Urostomy evaluation

Urostomy patency assessment was performed weekly by clinical evaluation. Patency was maintained if we found urine leakage from urostomy. In case of any doubts, a paper towel was applied to the urostomy. A dry towel confirmed the stoma closure. Simultaneously, the stoma’s appearance, the possible prolapse of the conduit, and the postoperative wound were evaluated. Animal observation time was carried out until a completely closed urostomy was noticed. Ultrasound (Toshiba TUS-X100) of the urinary tract was performed weekly. Blood tests were also randomly performed on the day of surgery, 1 and 2 months after surgery, and on the day of euthanasia. The ureters and conduits’ patency were assessed by computed tomography (Toshiba Astelion CGGT-028A model) via central line intravenous contrast injection, diluted iompromide 1:1 Ultravist 300 NaCl 0.9% solution, month after reconstruction and just before the end of observation. Due to the urostomy closure, the second CT examination did not take place in some cases. Random CT evaluation after 7–8 weeks post reconstruction procedure in each Group was performed. Percutaneous pyelogram with diluted 1:1 Ultravist 300 iompromide with saline solution with range 40–50 cm H_2_O pressure were evaluated. The pyelogram was collected by the X-ray machine Polyrad S, Poly-S-FMTS Model.

### Histology evaluation

An overdose of intravenous phenobarbital administration sacrificed the animals. The tissue material was fixed in 10% buffered formalin for 24 h. Representative tissue sections were placed in histopathological cassettes and processed in the tissue processor. The material was gradually dehydrated in a series of ethyl alcohol with increasing concentrations (80.0–99.8%), subsequently cleared in the xylenes series, and embedded in paraffin. The formed paraffin blocks were cut using a rotary microtome (Accu-Cut SMR 200, Sakura) into 5 μm paraffin tissue sections, placed on slides, and stained with H&E and Masson using the routine technique. The consecutive 3 µm thin tissue sections were cut and used for immunohistochemical analysis. The immunohistochemical staining was performed automatically in DAKO AUTOSTAINER Link 48 (Dako, Denmark). The immunohistochemical procedure started with the antigen retrieval in PT LINK (Dako) using EnVision FLEX Target Retrieval Solution. High pH, subsequently, endogenous peroxidase activity was blocked using Peroxidase Blocking Solution (Dako) for 10 min.

The antigen retrieval in PT LINK (Dako, Agilent Technologies) using EnVision FLEX Target Retrieval Solution, High pH, subsequently endogenous peroxidase activity was blocked using Peroxidase Blocking Solution (Dako, Agilent Technologies) for 10 min. Furthermore, tissue sections were incubated with primary (ready to use) antibodies: mouse monoclonal anti-CD31 (cat. No.: IR610, (Dako, Agilent Technologies)), mouse monoclonal anti-p63 (cat. No.: IR662, (Dako, Agilent Technologies)); rabbit polyclonal anti-S100 (cat. No: IR504, (Dako, Agilent Technologies)), mouse monoclonal anti-SMA (cat. No.: IR611, (Dako, Agilent Technologies)) for 20 min in RT (room temperature). The antibody complex was detected using EnVision Flex Anti-Mouse/Rabbit HRP-Labeled Polymer (Dako, Agilent Technologies) for 20 min in RT and localized using 3–3′diaminobenzidine (DAB) as the chromogen. Furthermore, the sections were counterstained with hematoxylin, dehydrated in increasing ethyl alcohol grades, cleared in xylenes, and mounted. Histology evaluation by two independent pathologists was assessed.

### Statistic evaluation

Results were presented as mean ± standard deviation (SD). The urostomy evaluation (patency, prolapse, and closure) was evaluated by the ANOVA method. Tukey’s multiple comparisons test assessed the significance of changes in the studied parameters in time. Differences between groups were considered significant at p < 0.05.

### Ethical approval

Ethical approval to report this case was obtained from The Polish Local Ethics Committee in Bydgoszcz, Poland (no. 43/2018).

### Statement of human and animal rights

All procedures in this study were conducted in accordance with The Polish Local Ethics Committee in Bydgoszcz, Poland (no. 43/2018) approved protocols. This study was carried out in compliance with the ARRIVE guidelines.

### Statement of informed consent

No human subjects in this article and informed consent are not applicable.

## Results

### Immunohistological evaluation of preimplanted scaffold before reconstruction from the 3rd Group

**Figure 5:**
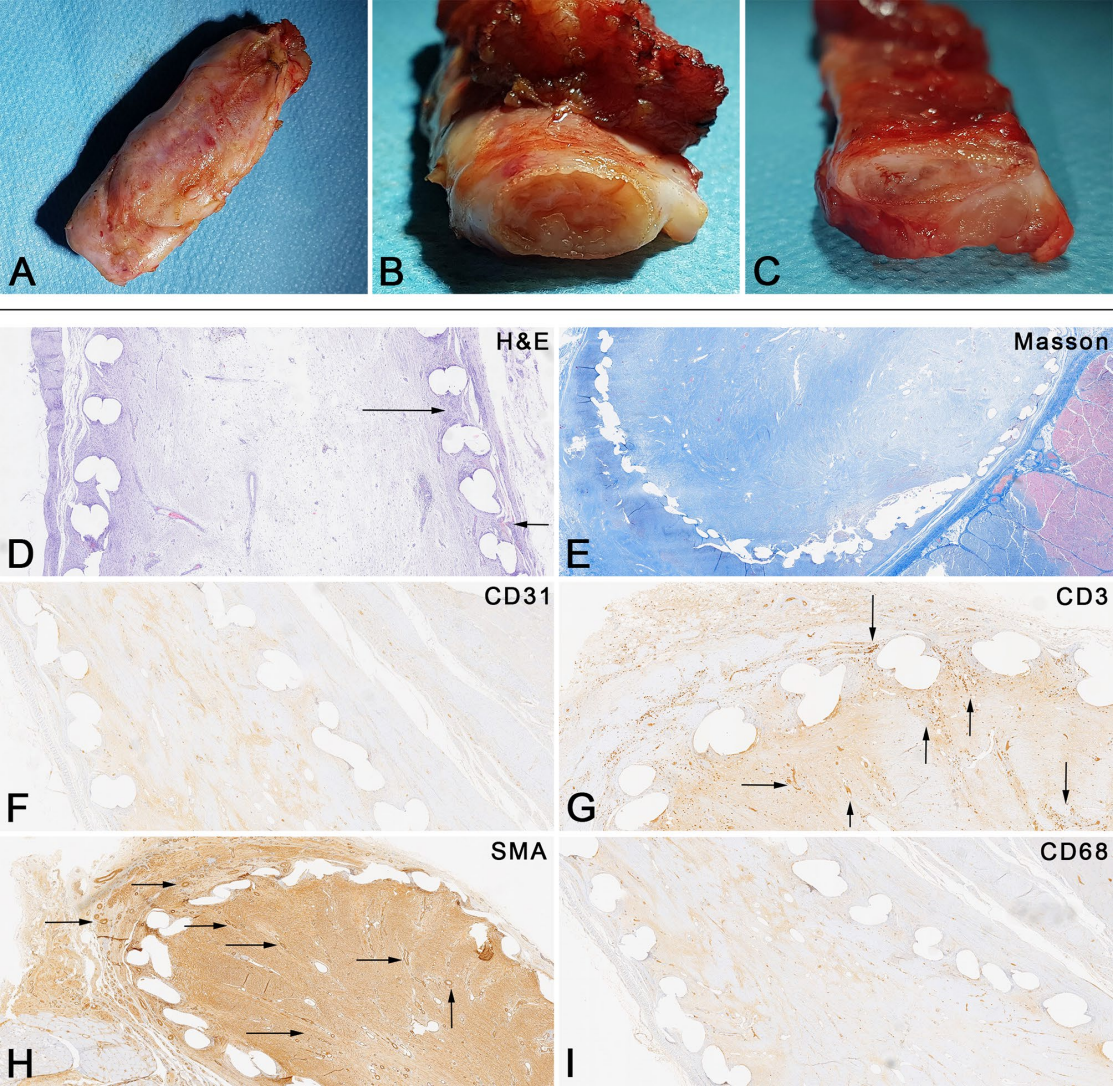
Macroscopic and immunohistological examination. (**A**–**C**) Macroscopic evaluation of preimplanted scaffold for histopathological evaluation, 1 month after intramuscular transplantation: (**A**) excised en block with adjacent tissue, (**B**,**C**) a cross-section of a conduit, we observed the fulfilled with connective tissue, it also confirmed the necessity of tube insertion before reconstruction for lumen preservation and reconstruction utilization. (**D**–**I**) Immunohistological assessment of implanted scaffold, 4 weeks post-transplantation. On (**D**) picture, we observed slight inflammation around the conduit with the formation of connective tissue—an arrow, (**G**) with arrows we marked the formed numerous blood vessels, (**H**) we staining of the smooth muscle of blood vessels confirmed neovascularization, (**I**) no chronic inflammatory infiltration (Hematoxylin and eosin-HE, Trichrome and Masson-Masson, CD31, CD3, Smooth Muscle Actin-SMA, CD68).

An inflammatory resorptive reaction was confined to the zone around the mesh fibers’ sites with the single presence of giant cells-“around foreign body” type. There is a slight infiltration of lymphocytes around the resorptive sites and single scattered eosinophils. The above changes are surrounded by abundant fibroblast proliferation with accompanying vascular proliferation. Inflammation (1/3), inflammation activity (0/3), resorptive reaction (1/3), vascular proliferation (3/5), fibrosis (4/5) (Fig. 5).

### In vivo assessment

**Figure 6:**
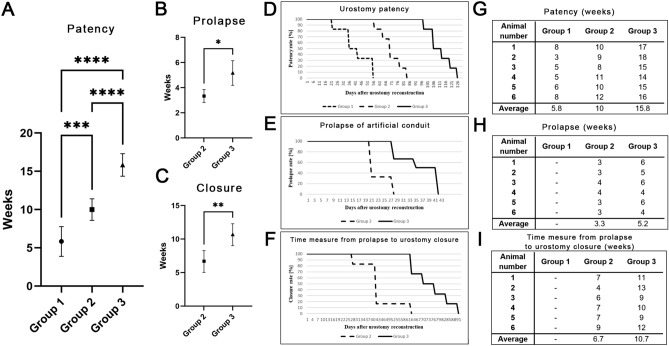
Urostomy evaluation in the presence of patency (**A**), prolapse (**B**), and closure (**C**). Points on the charts show the mean; error bars show the interquartile range. Statistical significance marked with: * < 0.01, **p < 0.02, ***p < 0.001, ****p < 0.0001. Kaplan–Meier patency (**D**), prolapse of artificial conduit (**E**), and time measure from prolapse to urostomy overgrown (**F**) curves after different methods of urostomy formation. (**G**–**I**) Data from individual animals from each group are summarized in tables.

All animals survived performed surgical procedures and observation period. We had two death during the induction of anesthesia, just before surgery. These animals were not included in this study. The postoperative observation time was conducted till the skin urostomy overgrown has been completed. The urinary flow through urostomy was presented in all animals just after surgery. The longest average patency was observed in the 3rd Group (preimplantation before reconstruction Group) 15.8 weeks compared to the 2nd Group (conduit without preimplantation) 10 weeks and the 1st Group (Control) 5.8 weeks with statistically significance p < 0.05. (Fig. 6). In one animal in a 1st Group, we observed ureteral catheter prolapse after 1.5 weeks after urostomy formation, resulting in occlusion within 3 weeks post-operation. The longest 18 weeks patency was observed in the 3rd Group. The urostomy functioned in the shortest time, 3 weeks in the 1st Group. The latest average prolapse was observed in Group 3 vs. Group 2 (5.2 vs. 3.3). We evaluated the time measured from conduit prolapse to closure urostomy (weeks). Time measures from prolapse to functionless urostomy in the 2nd vs. 3rd Group differed from each other: (6.7 vs. 10.4) respectively (Fig. 5).

Hydroureteronephrosis was confirmed in all examined groups without noticeable differences between the groups. In all groups, macroscopic assessment of affected kidneys showed a 40% increase in kidney size in the 1st Group and 50–55% in the 2nd and 3rd Group. The histological evaluation revealed a loss of kidney structure—significantly widened renal pelvis with flattened medulla and cortex. The loss of nephrons was observed during microscope evaluation. Moreover, the infiltration of inflammatory cells was characteristic of pyonephrosis. Renal changes were similar in all groups. The operated ureters were significantly widened compared to the unoperated ones. Furthermore, we found the most severely dilated ureters in the 3rd Group than in the 2nd Group and 1st Group compared to control ones (125% vs. 106% vs. 80%). No incidence of stone formation nor encrustations was observed.

### Histology assessment

**Figure 7:**
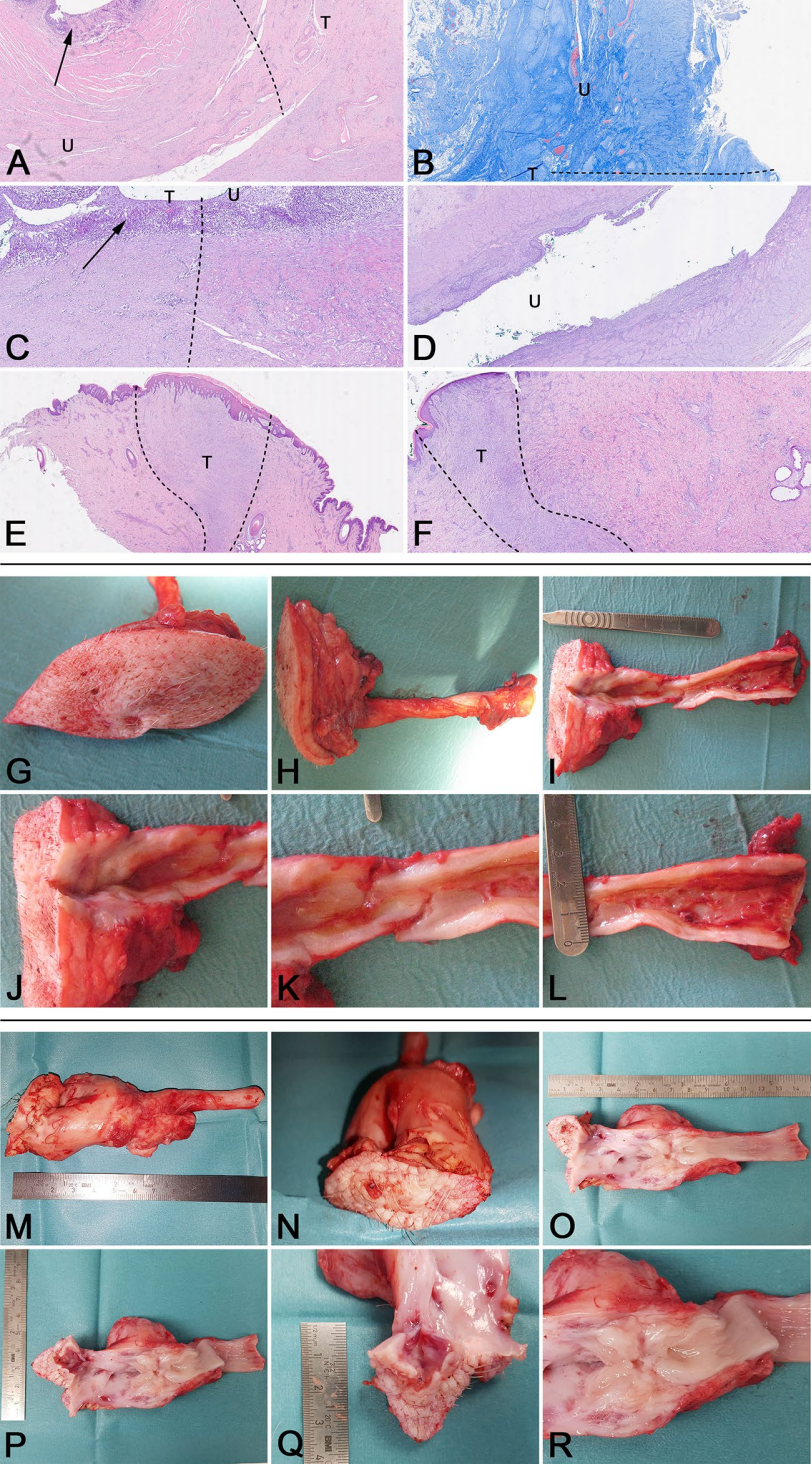
Histological evaluation and macroscopical examination. Samples assessment at the end of 24 weeks of observation. (**A**–**E**) Preimplanted Group 3: (**A**–**C**) ureteral anastomosis (junction between tube formed after conduit and ureter—dotted line), with arrow we marked disrupted urothelium both ureter and in a tube, (**D**) represent a destroyed ureter structure after reconstruction, (**E**) a completely urostomy overgrown, (**F**) a completely overgrown urostomy in a Group 2, the most intense inflammatory cells infiltration in the canal itself and the skin surrounding tissues. Ureterocutaneostomy in Group 1, 6 months after reconstruction. The closure of the stoma within the skin is unambiguous (**J**). Moreover, we observed distended, ureter—marked with a scalpel shaft (**K**) and shown in the photo (**L**). Pictures were taken at the end of 24 weeks of observation after a urinary diversion in a pretransplant conduit before reconstruction—Group 3. A narrowing accompanied the atresia of the skin canal in the conduit’s junction with the ureter with connective tissue growth (**O**,**R**). More than a significant widening of the ureter to over 2 cm with its post-inflammatory destruction (**P**).

In all animals terminating the observation, the urostomy was closed. It has been confirmed by histopathological evaluation. The connective tissue filling the stoma was epithelialized in all animals. In the histopathological evaluation, the tunnel after the prolapsed conduit was very similar to the damaged, dilated ureter in the 1st Group. No calcification was observed in the urinary tract. Kidney evaluation showed extreme parenchyma destruction due to hydroureteronephrosis (Fig. 7).

### Ultrasound examination

Systematically repeated ultrasound examinations showed a widening of the renal pelvis along with systematically prolapsing of the urostomy. In the artificial both not and preimplanted groups, we observed a presence of fluid around the conduit. In the 2nd Group, due to AC leakage, the first incidence of fluid appearance around the conduit was observed 1-week post-implantation. However, in the preimplantation before reconstruction (3rd Group), the first fluid sleeve formation was presented between 3 and 4 weeks after urostomy formation (Fig. 7).

### Retrograde pyelogram

**Figure 8:**
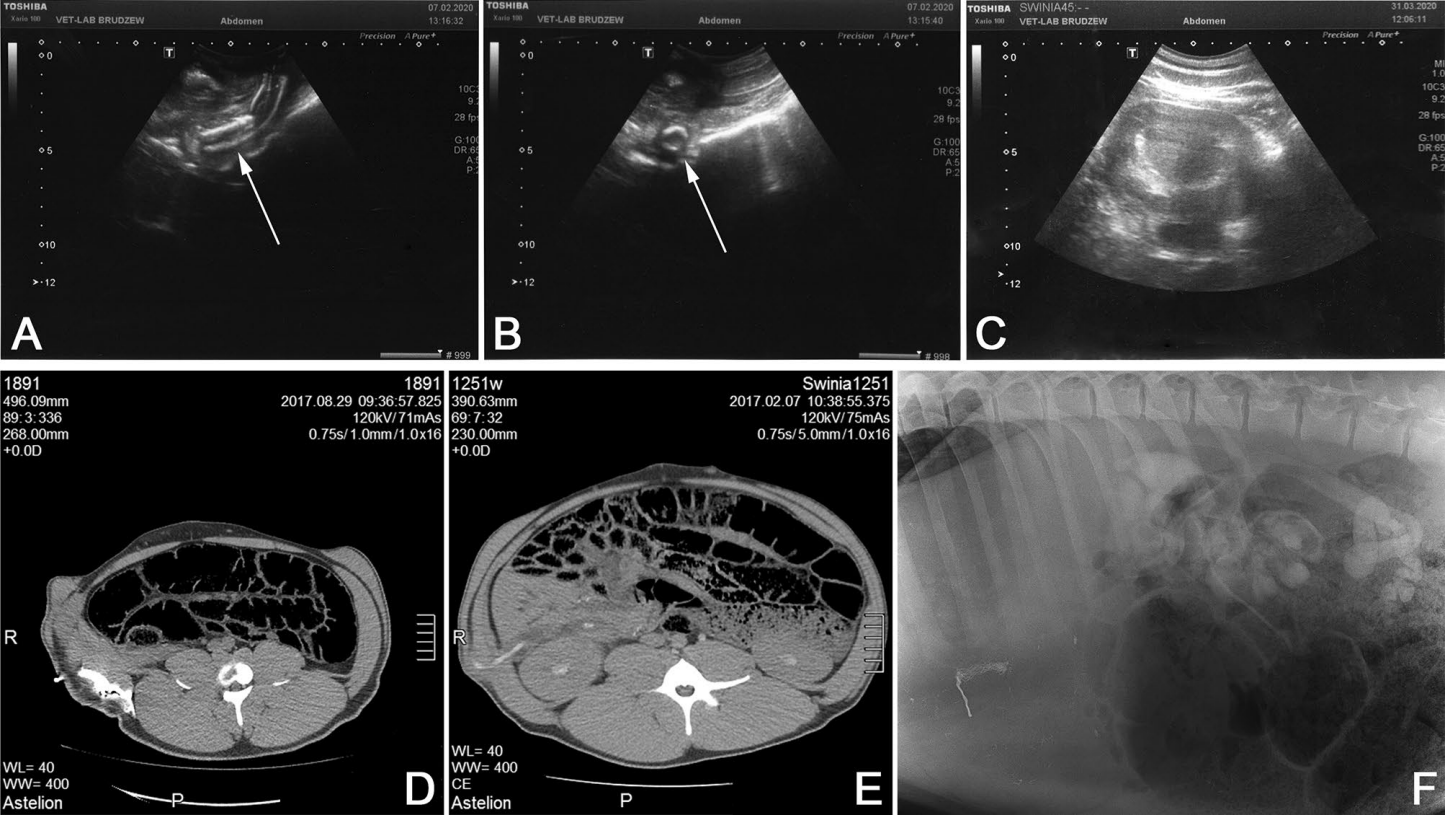
Results of USG and computed tomography analysis. USG evaluation after 4 weeks of post-reconstruction procedure in the preimplanted conduit Groups (**A**,**B**). We can observe the junction between the preimplanted conduit with a dilated ureter in the longitudinal section after 4 weeks post reconstruction procedure in the implanted group (**A**). Cross-section projection revealed the urine gathering around the conduit (**B**). Ultrasound abdomen examination revealed the widened calyces-pelvic kidney system in a control group after 7 weeks post ureterocutaneostomy. The result of computed tomography with contrast performed to assess the patency of the reconstructed ureter with an implanted conduit: (**D**) examination 7 weeks after the reconstruction of the ureter with the preimplanted conduit (Group 3)—the outflow of contrast through the conduit were visible. (**E**) Group 2—condition after ureter reconstruction with conduit 8 weeks after reconstruction—contrast outflow confirms urostomy patency. (**F**) We present an animal’s pyelogram in 17 weeks of observation preimplanted conduit-Group 3. The intra-pelvic contrast administration showed a significant widening of the calyces-pelvic renal system and the ureter’s lumen itself with no contrast outflow. Moreover, in all groups, the constriction and closure of the ureter occurred at the skin level.

Pyelogram confirmed stenosis in a skin area with significantly widened ureters, especially in a 3rd, second, and 1st Group. We did not notice stenoses between the conduit and the ureter in both 2 and 3 Groups (Fig. 8).

## Discussion

Even though urinary diversion’s intestinal segment is a gold standard, there are no effective reconstruction methods without opening the gastrointestinal tract^18,19^. Most experimental studies utilized an AC covered with degradable materials for inducing integration and urine separation^20^. However, the use of these resorbing materials causes a different inflammatory reaction similar to absorbable surgical sutures^21,22^. Our study aimed to use propylene mesh’s regenerative potential to form tubular connective tissue-a 3D construct^23^. Of course, the main disadvantage of clinical application is the need to perform an additional preimplantation procedure. The clinical application’s subsequent surgical procedure can be minimized by performing the procedure under local anesthesia—2 or 3 weeks before radical cystectomy.

Another question arisen was where is the best preimplantation localization. Using our knowledge and Korossis et al.^24^, the best method for conduit preimplantation is the abdomen cavity comparing to subcutis or intramuscular implementation^25,26^. Intraperitoneal environment, greater omentum, quarantines excellent blood supply, which implicated sufficient tissue biocompatibility^27^. The main limitation is the need for general anesthesia and laparotomy complications. However, Basu completed the preclinical trials with the omentum utilization and entranced the clinical part, but there are no reports^15^. Only Sloff pre-implanted the conduit but removed it from the implantation region during urinary tract reconstruction^16^. This procedure depleted the vasculature system. Contrary to this, we preimplanted the conduit in a final destination site to guarantee proper conduit vascularization (Fig. 6D,E).

Till now, all conducted studies have assessed the patency of the canal regardless of whether the conduit was present or not Anke et al.^28^. What makes our work different from others is the reliable assessment of the time from the conduit’s prolapse to the complete closure of the urostomy. In the preimplantation Group, we found out that the function of the conduit lasted the longest time and was related to the latest urostomy obliteration^28^.

Until now, no study has looked at the angle position of the conduit to the intra-abdominal pressure. The appropriate deviation of the AC from the perpendicularly operating abdominal cavity pressure will guarantee no mechanical pushing of the conduit outwards. This allows for the preservation of new vessels formed around the conduit in the regeneration phase.

Some authors argued that polypropylene mesh is tissue incompatible^21^. If it were true, no one would use it in hernias treatment^29^. The material is one of the few that can be used as a readily available and inexpensive product.

So far, all published studies have assessed the outflow of urine from the ureterocutaneostomy as the most crucial factor in AC function assessment. Moreover, it is not easy to evaluate the conduit function if the ureteral catheter is inserted for 3 weeks, and the observation period is 4 weeks, as the Geuties published^14^. It is only 1 week of observation time without a catheter. Moreover, in our opinion, the patency and urine outflow does not coincide with the secretory function of the kidney, which can be significantly impaired after reconstruction. We found a significant delay of contrast excretion produced after 2 weeks of catheter removal, despite presented satisfactory urine outflow.

The collation ureterocutaneostomy control group allowed us to observe mechanisms that potentially impact the patency of the urostomy. The simultaneous reconstruction of the ureter with a tube caused connective tissue tunnel occurrence due to infection and urine toxicity (Group 3). These two factors induce the connective tissue canal formation with concomitant AC prolapse^30^. Later, the canal was lined with urothelium. The entire cascade processes involved in wound healing are responsible for successive urostomy overgrown. Inflammation, urine irritation causes constriction of the skin and subcutaneous tissue.

Skin epithelial stroma cells transmit acceleration signals to transform the urothelium into the squamous epithelium what accelerates overgrown^31^. Granulation and subsequent epithelialization progress very dynamically the smaller the urostomy are.

A question has been arisen by Sloff et al.^32^: should the control group not be the intestine continent diversion model? It is a proven model widely used in clinical practice, so the use of animals is unreasonable. Moreover, this method has been preclinically well evaluated^33–36^. Clinical problems with ureterocutaneostomy—the worse alternative for intestinal supplies of ureterostomy—and the lack of functional evaluation in a large animal model prompted us to use the ureterocutaneostomy as a control group^5^.

The conduit preimplantation before reconstruction temporarily separates the urothelium from the skin epithelium in the 3rd Group. The difference in the length of patency between the Group with the preimplant conduit before reconstruction in the 3rd Group and the not preimplanted conduit (2nd Group) follows directly from the resistance of ascending infection and toxic effects of urine. Only the preimplanted Group (3rd Group) had this biological barrier against these unfavorable factors, directly translated into longer canal patency. After the AC prolapsed in the 3rd Group, the situation resembles the beginning observed in Group 2. The slower lining of the urothelium canal and the urostomy diameter resulted in slower overgrowth of the canal in the second and 3rd Group.

Notwithstanding cutting the ureter from the bladder, we eliminate partial autonomous nervous fibers controlling the process of urine transportation^37^. Distal ureter denervation disturbs the urine flow throughout the system. The effect of impaired distal systolic function multiplies the urine stagnation and then infection^38^.

There are two leading causes of this situation: ascending infection and toxic effects of urine. Why an AC preimplantation? Because the resulting connective tissue creates a partial barrier before urothelium migration that separates the synthetic material from urine and ascending infection. These two mechanisms are responsible for the main cause of failure when fabricating a urostomy with artificial materials occurs.

The first tissue-engineered Neo-Urinary Conduit (NUC) clinical trial (NCT01087687) for patients after total cystectomy was conducted by^39^. Eight patients underwent successful NUC reconstruction in the UD model. The average NUCs lifespan was 250 days (from 42 to 484 days). The NUC reconstructed patients had 3/6 stenosis and 3/6 strictures. This study proved that the preclinical model did not recapitulate the human condition, similar to our findings. Furthermore, they identified three critical issues for the successful implementation of AC in the UD model: dedicate scaffold construction, ideal cell types, and AC nutrition supply^39^. Although the preimplanted AC meets these assumptions: material is stiff and has no tendency to collapse, lack of cells is replaced by connective tissue sleeve, and blood supply delivers from the adjacent tissues, the results are not spectacular.

## Conclusions

In our opinion, a preimplanted artificial conduit’s essential feature is the ability to create a partial barrier between tissues and urine. It is a prerequisite for conduit integration and thus for long-life urostomy patency.

## Data Availability

All data generated or analyzed during this study are included in this published article.
